# The future of PFA in cardiac ablation procedures

**DOI:** 10.1093/europace/euag210

**Published:** 2026-10-21

**Authors:** Jason G Andrade, Samuel J Asirvatham, Mélèze Hocini, Peter Karl Jacobsen, Nico Reinsch, Domenico G Della Rocca, Andrea Sarkozy, Nicholas Tan, Jason A Tri, Claudia Auricchio, Giovanni Raciti, Tomás García-Sánchez, Jacob Koruth

**Affiliations:** Department of Medicine, Vancouver General Hospital, Vancouver, Canada; Department of Cardiovascular Medicine, Mayo Clinic, Rochester, MN, USA; Department of Pediatric and Adolescent Medicine, Mayo Clinic, Rochester, MN, USA; Hôpital Cardiologique du Haut-Lévêque, CHU de Bordeaux, Bordeaux-Pessac, France; IHU LIRYC (L'Institut de Rythmologie et Modélisation Cardiaque), Université de Bordeaux, Bordeaux-Pessac, France; Arrhythmia Center for the Capital Region of Denmark, Rigshospitalet and Gentofte Hospital, Copenhagen, Denmark; Department of Electrophysiology, Alfried Krupp Hospital, Essen, Germany; Department of Medicine, Witten/Herdecke University, Witten, Germany; Heart Rhythm Management Centre, Postgraduate Program in Cardiac Electrophysiology and Pacing, Universitair Ziekenhuis Brussel—Vrije Universiteit Brussel, European Reference Networks Guard-Heart, Brussels, Belgium; Heart Rhythm Management Centre, Postgraduate Program in Cardiac Electrophysiology and Pacing, Universitair Ziekenhuis Brussel—Vrije Universiteit Brussel, European Reference Networks Guard-Heart, Brussels, Belgium; Department of Cardiovascular Medicine, Mayo Clinic, Rochester, MN, USA; Department of Cardiovascular Medicine, Mayo Clinic, Rochester, MN, USA; Medical Education—Boston Scientific Corporation EMEA, Madrid, Spain; Medical Education—Boston Scientific Corporation EMEA, Madrid, Spain; Medical Education—Boston Scientific Corporation EMEA, Madrid, Spain; Helmsley Electrophysiology Center, Mount Sinai Fuster Heart Hospital, Icahn School of Medicine at Mount Sinai, New York, USA

**Keywords:** Pulsed field ablation, Future, Waveform, Catheter design, Electroanatomical mapping, New patient groups, Terminology, Standarization, Clinical trials

## Abstract

Pulsed field ablation (PFA) has rapidly emerged as a major development in catheter ablation, with increasing clinical adoption driven by procedural efficiency and a favorable safety profile compared with thermal energies. This article summarizes the future directions of PFA in electrophysiology, focusing on technological innovation, and how these advances are expected to expand PFA clinical indications. Additionally, this article provides a perspective on current gaps and future challenges of PFA. A perspective on future developments in new waveforms, catheter architectures, and integration with electroanatomical mapping systems for PFA is summarized. The document discusses novel waveform configurations and catheter design concepts that may improve lesion quality, procedural versatility, and safety. Additionally, this article also reviews how parallel advances in electroanatomical mapping integration, artificial intelligence, and robotic navigation are expected to enhance procedural guidance and enable individualized therapy. This manuscript summarizes the potential applications of PFA beyond pulmonary vein isolation and how emerging evidence supports its feasibility for linear atrial lesions, substrate modification, treatment of complex atrial tachycardias, and ventricular arrhythmia ablation. In addition, this article provides insights on how PFA may facilitate catheter ablation in new patient populations including elderly patients and those with higher procedural risks. Finally, this manuscript provides an outlook at major remaining challenges and knowledge gaps for PFA. The lack of homogenous procedural endpoints definitions and lesion durability assessment are discussed. Furthermore, this article highlights the need for standardized terminology and reporting in an environment where the high heterogeneity between available commercial systems complicates comparison across technologies.

## Graphical Abstract

**Graphical Abstract:**
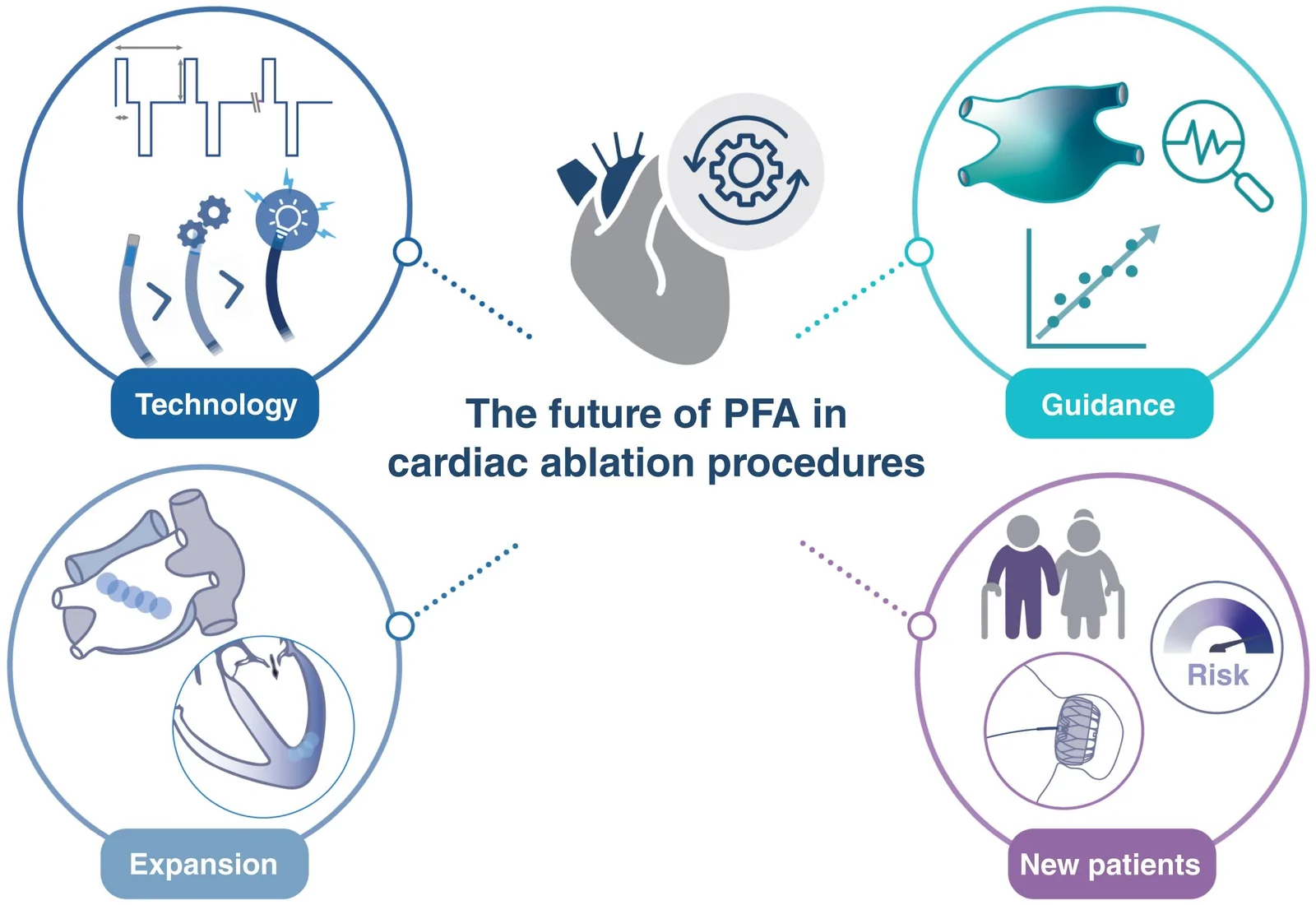

## Introduction

Pulsed field ablation (PFA) has undergone exceptionally rapid development in recent years, transitioning from an experimental energy source to a clinically established modality in cardiac electrophysiology. PFA has generated considerable interest as an alternative to conventional radiofrequency and cryothermal ablation. The rapid widespread clinical use has been accompanied by an unprecedented technological innovation, with multiple dedicated platforms now available and large-scale trials rapidly defining its role in atrial fibrillation treatment.

This article provides an overview of the future perspectives of PFA in cardiac electrophysiology. It first examines the potential technological evolution of PFA with a focus on new waveforms, advances in catheter design, and procedural integration with 3D mapping systems. Subsequently, the article explores new and emerging indications, highlighting the progressive expansion of PFA beyond pulmonary vein isolation (PVI) in atrial fibrillation to more complex arrhythmia substrates and broader clinical applications with a specific focus on future applications in the ventricle. Additionally, new patient groups benefiting from PFA are examined. Finally, the article addresses the remaining scientific and clinical challenges associated with PFA, including remaining knowledge gaps, standardization of protocols and reporting and discussing the current challenges for system comparison. Finally, an outlook about ongoing and future clinical trials is provided. With the remarkable innovation pace in PFA, the aim of this article is to provide a structured synthesis of key directions for future developments, research and clinical adoption of this disruptive ablation technology.

## Technological evolution of PFA: shaping energy delivery and guidance

### New waveforms

From the early years of PFA exploration, there was already some awareness that waveform design would have a major influence on lesion creation as well as safety and procedural workflow. The early development of the first PFA system initially explored the use of a monophasic square waveform; although this was effective in isolating the pulmonary veins, prominent skeletal muscle stimulation was noted, which compelled the use of general anaesthesia and paralytics.^1^ Transition to biphasic waveform delivery for the second half of the PEF CAT study^2^ showed how skeletal muscle activation was noticeably reduced, thereby making deep sedation a feasible option for operators. Subsequent studies showed that biphasic PFA may also have other additional safety advantages over monophasic PFA; in a porcine study, monophasic PFA delivered on the T wave of the cardiac cycle consistently triggered ventricular fibrillation, whereas this was not seen with biphasic delivery.^3^ Nowadays, most contemporary clinical platforms deliver groups of biphasic square wave pulses with individual pulse duration in the short microsecond range.

As our knowledge of PFA continues to mature, other aspects of waveform design are under active investigation. Specifically, other pulse durations beyond the microsecond duration domain are under study (see *Figure 1A*). In preclinical and early first-in-human studies, sub-microsecond PFA in the nanosecond range has been shown to be effective in creating transmural myocardial lesions in the atrium and pulmonary veins with both endocardial as well as surgical approaches.^4–6^ Additionally, exploratory large animal studies have also demonstrated the feasibility of nanosecond PFA for ventricular myocardial ablations.^7–9^ Effects on cells have been studied since the early 2000s suggesting specific effects on intracellular structures and demonstrating their ability to induce electroporation of intracellular structures.^10,11^ From a clinical perspective, nanosecond PFA may induce skeletal muscle contraction to a lesser extent than microsecond PFA, which may widen options for intraprocedural sedation. Nanosecond pulses could require considerably increased voltage levels delivered by the PFA generator potentially involving technological challenges regarding isolation and impedance matching for the correct pulse transmission to the target tissue. Additionally, the effects of submicrosecond pulses may substantially differ between purely nanosecond pulses of a few nanosecond duration to submicrosecond pulses in the order of hundreds of nanoseconds. Other ongoing uncertainties that will require further study include the extent of microbubble formation and long-term lesion durability.

**Figure 1:**
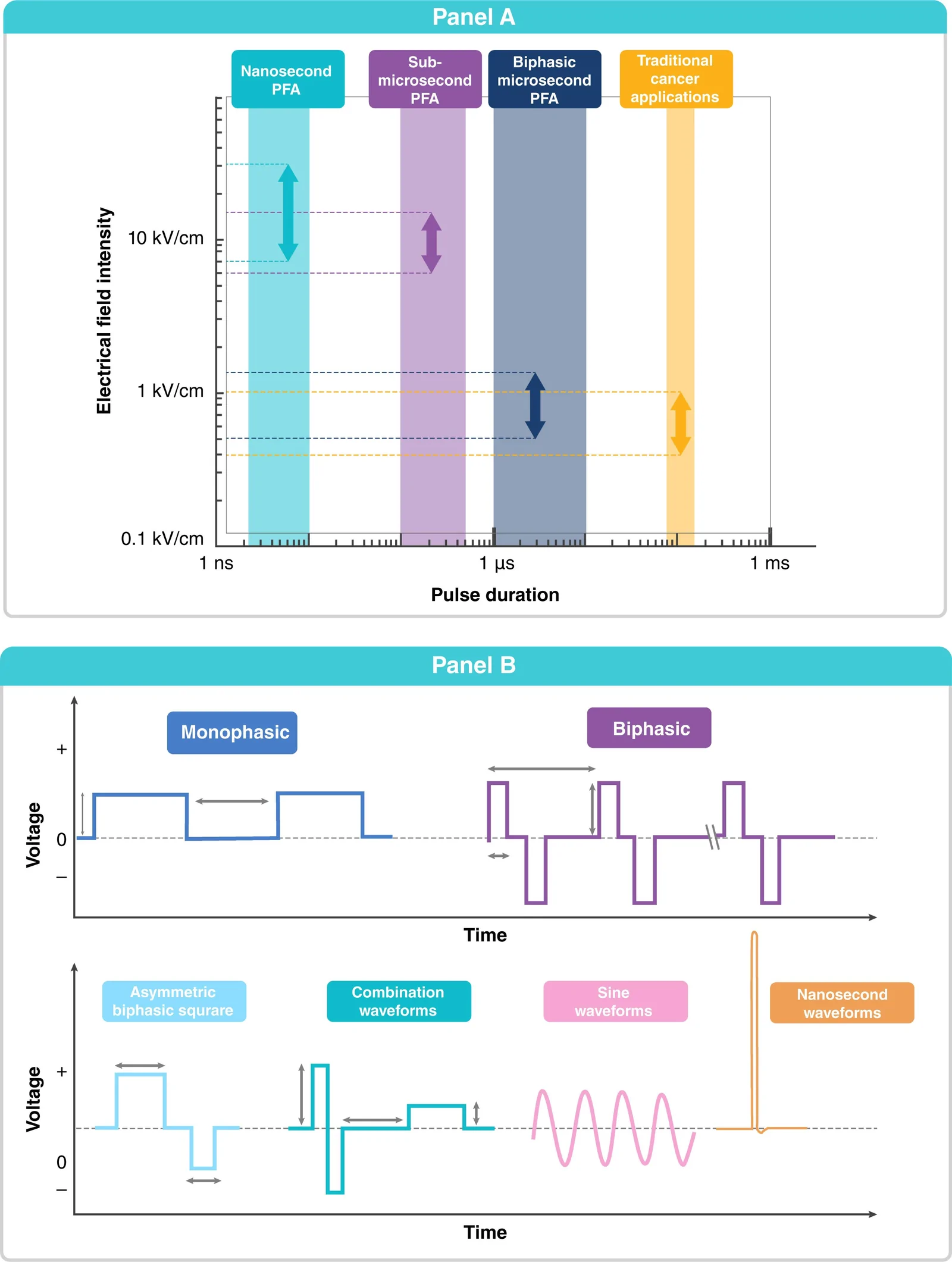
*A*) Summary of main pulse duration domains for irreversible electroporation applications. *B*) Summary of the main waveforms explored for PFA applications.

Besides pulse duration, future developments may involve using alternative waveforms different from biphasic square pulses or even combinations of different pulse types (see *Figure 1B*). The use of sine waveforms has been investigated as an alternative means of PFA delivery. Preclinical chronic porcine data demonstrated durable PVI and linear lesion formation without clinically significant pulmonary vein stenosis, detectable microbubbles, electrical arcing, thrombus, charring, or major collateral injury.^12^ In the BURST-AF study, coherent sine-burst electroporation achieved 94.1% per-vein durability, 94.4% durability of additional posterior wall and cavotricuspid isthmus lesions, and 92.6% freedom from atrial arrhythmias at 6 months.^13^ Additionally, altering the symmetry of biphasic square waveforms, different amplitudes or durations between the positive and the negative parts of the biphasic pulse, has also been explored; in a pilot study utilizing an asymmetric biphasic square waveform (positive phase 5 µs, negative phase 3 µs), successful PVI was achieved in 10 patients, with no atrial arrhythmia recurrences or complications over 6 months.^14^ Other pulse parameters including interpulse delay may be important determinants of lesion creation as well.^15–17^

In summary, there is a plethora of PFA parameters that factor into ablation efficacy and safety; furthermore, waveform delivery parameters must be considered in context with the ablation catheter that is being used. It is therefore contingent upon the medical community and industry to be aware of the complexities and nuances behind these parameters and to ensure systematic testing prior to clinical implementation.

### Next-generation PFA catheters: optimizing energy delivery

Besides new developments on PFA waveforms, the second most important element allowing PFA evolution is the catheter design. While some contemporary PFA systems are based on repurposed catheter designs, next-generation PFA catheters are expected to evolve to purpose-built PFA designs incorporating features that should address current limitations in efficacy, precision, side effects and workflow efficiency.

A central concept in future catheter development is the device form factor and the associated tissue surface area treated during a PFA application. Contemporary PFA systems include small-focal, large-focal, regional and single-shot platforms adapted for focal, linear or wide-area lesion creation or for circular PVI.^18^ Regarding single-shot devices, while current systems already enable rapid PVI, the realization of a ‘true single-shot’ device, one application-one position, remains a current design challenge. Furthermore, anatomical variability and complex arrhythmogenic substrates support the need for adaptable and multifunctional designs capable of adapting to diverse scenarios and anatomies. Next-generation catheters capable of dynamically adopting multiple shapes to provide large area or focal ablation functionalities would represent a paradigm shift.

Another potential area of innovation is precise electric field control for shaping lesion size and limiting collateral effects. Although PFA preferentially ablates cardiac tissue, electric field propagation beyond the target region, particularly towards near sensitive structures, can produce undesired collateral effects like excessive neuromuscular stimulation and associated pain or coronary spasm. Advancements in electrode configuration allowing electric field confinement or electric field shielding strategies could enable a more spatially confined ablation minimizing unintended effects on nearby tissues.^19^

Fabrication materials, especially electrode materials, are another area of potential future innovation where traditional metallic electrodes may be supplemented or replaced by novel conductive polymers that offer enhanced flexibility, improved tissue contact, and superior biocompatibility. Parallel to material innovation, fabrication techniques are likely to undergo a transition from manual assembly to scalable manufacturing processes such as printed electronics and additive manufacturing. These approaches will allow precise control over electrode geometry and enable the integration of complex electronic and sensing components directly into the catheter structure. Multifunctional electrodes capable of simultaneous sensing and energy delivery could represent an advantage in future designs.

With the expected evolution of software tools, future PFA catheters are also expected to incorporate ultra-high-density electrogram recording capabilities. Enhanced signal quality and spatial resolution could improve procedural outcomes and reduce arrhythmia recurrence rates.^20^ Beyond electrograms, the integration of multiparametric sensing—encompassing temperature, impedance, contact, force, optical information or even tissue metabolic activity—could provide comprehensive real-time feedback on tissue status during ablation.

An additional area where improvements in catheter architecture may be implemented is eliminating the need for electrode irrigation. Although PFA relies on a non-thermal mechanism for tissue ablation, irreversible electroporation, the application of high pulsed electric fields on biological tissues can induce considerable short-duration rises in temperature by the Joule heating effect. PFA system optimization should ensure that the thermal profile of any PFA platform does not exceed the thresholds for thermal damage. Accordingly, some contemporary systems require considerably high electrode irrigation rates to ensure lesion formation without unintended thermal effects.^21^ Future improvements in waveform design and purpose-built catheters could overcome the need of irrigation generalizing irrigation-free catheter designs.

The convergence of these technologies with artificial intelligence (AI) and robotic systems may further transform future catheter-based interventions. AI algorithms could support operators by optimizing pulse delivery and guiding optimal catheter positioning. Lastly, although robotic navigation is not a new concept,^22^ the rapid advancements in robotic systems may enable this concept rapidly evolving towards a future of semi-automated or fully automated ablation procedures.

Finally, sustainability considerations are becoming increasingly relevant.^23,24^ The development of reusable or partially reusable catheters, along with environmentally conscious manufacturing processes, could reduce medical waste and overall healthcare costs without compromising safety or performance.

### The role of mapping with PFA: future guidance on PFA procedures

Initial implementations of PFA technology did not include dedicated integrations of the ablation catheter into the electroanatomical mapping systems, and procedures were guided by traditional imaging techniques, mainly visualization of the catheter under fluoroscopy or rudimentary integration into mapping systems. Nowadays, although some systems are still extensively used without the support of electroanatomical mapping, the rapid development of PFA has led most contemporary platforms to incorporate dedicated, purpose-built catheter solutions specifically designed for PFA delivery.

As optimal clinical implementation of PFA relies heavily on accurate and reproducible procedural guidance, electroanatomical mapping is expected to play an increasingly important key role in future developments.^25,26^ The integration of electroanatomical mapping into PFA workflows should focus on several key domains, including accurate catheter positioning within complex cardiac anatomies, reliable assessment of catheter–tissue interaction, robust evaluation of lesion formation, and comprehensive characterization of the underlying substrate, among others.^27^ Mapping technologies may evolve from a primarily navigational role towards a more active contribution to real-time decision-making and individualized ablation strategies—an evolution that is likely to become even more relevant as the clinical indications for PFA expand toward increasingly complex atrial and ventricular substrates.

Accurate catheter positioning represents a foundational element in the role of mapping in PFA, as PFA efficacy is highly dependent on catheter position and orientation, spatial consistency, and anatomical alignment within complex cardiac structures.^28,29^ Ablation efficacy is determined by the intersection of the target tissue with the volume where the electric field intensity exceeds the lethal threshold for cardiac tissue specific for each PFA system. Future developments are likely to include high-accuracy anatomical acquisition tools, improved modelling tools for accurate depiction of the predicted PFA field distribution, or integration of intracardiac echocardiography for more precise catheter rendering—particularly for non-linear and multi-spline designs. In addition, improved visualization of sheaths and guidewires may contribute to further reductions in fluoroscopy usage.

The assessment of catheter-tissue interaction constitutes a complementary aspect of procedural guidance beyond spatial positioning alone. Although the value of traditional contact force metrics is still under study and may not provide additional benefits for every PFA design, effective PFA lesion formation will be optimized by minimizing the distance between the catheter and myocardial tissue.^30^ Future mapping implementations are therefore expected to focus on the refinement and validation of PFA-specific indicators of tissue interaction, such as impedance-based parameters, providing real-time feedback on contact quality and enabling operators to optimize energy delivery without relying on paradigms derived from thermal ablation technologies.

Defining robust lesion assessment strategies and procedural endpoints remains one of the most significant challenges in the broader adoption of PFA. Conventional markers of ablation efficacy, including acute voltage reduction or immediate conduction block, may not fully reflect the unique temporal and electrophysiological effects associated with PFA lesions.^31^ Electroanatomical mapping is therefore expected to play an increasingly important role in interpreting lesion formation and distinguishing transient from durable effects. In this sense, future systems may include optimized real-time algorithms able of analysing and extracting electrogram features such as morphology and recovery dynamics of unipolar electrograms that could help predict areas of reversible or irreversible electroporation after pulse delivery.^32–35^ Another important element that may be integrated into future mapping systems relates to the ability for energy delivery titration. Nowadays, PFA lesion formation is governed by procedural workflow optimization rather than energy delivery titration. Although several PFA-specific indices integrating variables such as voltage, contact force, and application count have been proposed,^36–39^ the validity of their predictive performance is still to be proven in the clinical setting. Increasing understanding of PFA mechanisms may lead to the development of novel system-specific predictors that most likely will not only rely on traditional parameters used in thermal ablation but also on new concepts specifically developed for PFA.

Mapping systems also offer the potential to integrate substrate characterization into PFA decision-making, supporting a more individualized approach to ablation, particularly in patients with persistent atrial fibrillation. Although no individualized ablation strategy has yet demonstrated clear superiority over antral PVI alone,^40,41^ the increased procedural efficacy and safety profile associated with PFA may lower barriers to evaluating more advanced mapping-guided strategies. These improvements could include novel algorithms, AI–assisted analysis, or panoramic mapping techniques, among others. In this setting, mapping may contribute not only to procedural execution but also to strategic planning and patient-specific optimization of PFA therapy.

Continued integration of mapping-derived information into PFA procedures will be essential to establish reproducible standards, refine procedural endpoints, and support the safe and effective expansion of PFA across a broader range of clinical indications.

## New indications: expanding the clinical horizon of PFA

### Beyond pulmonary vein isolation: extending PFA to complex atrial arrhythmias

PVI remains the cornerstone of catheter ablation for AF. However, recurrence and the need for repeat ablation remain frequent, especially in persistent AF, prompting consideration of strategies beyond PVI to address non-pulmonary vein triggers and atrial substrate.^42^

PFA increases the procedural practicality of ‘beyond-PVI’ approaches. Energy delivery is rapid and repeatable, enabling efficient extension of ablation beyond PVI, such as linear lesion sets and targeted substrate modification (e.g. low-voltage areas).^43^ Myocardial preferentiality provides a rationale for broader substrate modification with a favourable collateral profile, supporting more extensive atrial lesion sets beyond antral PVI.^44^ Future expansion is likely to focus on defined atrial arrhythmia substrates and anatomical isthmuses. Typical flutter is a prime target for linear PFA at the cavotricuspid isthmus (CTI), with emerging data showing high acute block and procedural efficiency.^45^ For perimitral flutter, PFA may enable a more durable mitral isthmus block, with coronary sinus applications as a potential adjunct to address epicardial connections. Similarly, complex post-ablation atrial tachycardias (roof- and perimitral circuits and scar-related re-entry) may be addressed through mapping-guided linear sets and focal consolidation.^46^ Key limitations for these indications include defining robust procedural endpoints, ensuring chronic durability of linear block, and establishing adequate dosing workflows associated with lesions beyond PVI. Given the favourable safety profile, interest in extending ablation beyond PVI has increased.^47^

In parallel, platform evolution is lowering barriers to complex procedures. New catheter generations and system refinements increasingly combine PFA delivery with 3D mapping integration, enabling navigation and lesion deployment beyond antral PVI within familiar workflows. This availability of 3D-guided PFA broadens the technical scope for complex atrial arrhythmias, while iterative refinements in catheter design and platform integration aim to improve applicability and procedural consistency. As mapping-linked PFA is adopted more widely, incremental improvements in catheter design and software integration may support more consistent coverage of complex atrial substrates; the clinical impact, however, requires prospective validation.^48–50^

Device development is also diverging into complementary tool classes, which include single-shot multielectrode geometries (basket/flower and circular designs, including size-adaptable variants) and focal or large-footprint platforms.^51^ Single-shot designs are optimized for rapid, standardized antral coverage and can be redeployed to adjacent atrial regions—most commonly the left atrial posterior wall.^42,51^ Focal and large-footprint concepts support line completion and regional substrate modification under mapping guidance, particularly in redo procedures.^51^ Notably, lattice-based platforms have enabled reversible electroporation mapping with transient conduction block to help localize critical sites in re-entrant atrial tachycardias; early clinical experience suggests feasibility and reproducibility with moderate sensitivity, but further evaluation is required before broader adoption.^52^

Current pulse protocols have been primarily developed and validated for antral PVI, not for systematic beyond-PVI lesion sets. Proprietary pulse protocols limit cross-platform comparison and impede systematic optimization, but emerging directions include alternative pulse structures and delivery algorithms aimed at improving lesion homogeneity, lesion depth and procedural control.^51,53^ In selected settings, hybrid workflows with energy toggling (switching between PFA and radiofrequency) can be useful as a pragmatic bridge—for example, when completing depth-limited segments or closing residual gaps during early adoption and in anatomically challenging redo scenarios—but their role is likely to diminish as PFA catheter form factors, mapping integration, and dosing concepts for beyond-PVI targets mature.^53^

With continuing advances in catheter design, mapping integration, and energy delivery concepts, ‘beyond-PVI’ atrial ablation is moving further into focus and may improve long-term procedural effectiveness; however, robust long-term outcome data for systematic extension strategies remain limited.^40,41^

### PFA in the ventricle

Catheter ablation of ventricular arrhythmias (VAs) remains limited by several features intrinsic to ventricular substrate. Compared with atrial tissue, the ventricular myocardium is thicker, often scarred or infiltrated by fat, and may contain intramural or epicardial circuits that are difficult to reach with conventional radiofrequency (RF) ablation. In addition, VA ablation frequently requires treatment of extensive substrate, and procedural endpoints such as acute noninducibility do not always predict durable success. These limitations are particularly relevant in scar-related ventricular tachycardia (VT), in which inadequate lesion depth, incomplete transmurality, and inability to access protected substrate may all contribute to recurrence.

The main appeal of PFA in the ventricle is not simply novelty but the possibility of safer energy delivery with deeper scar penetration and reduced collateral injury relative to thermal energy delivery. Nonetheless, there are some key aspects that will require further clarification. PFA induces immediate electrogram (EGM) attenuation or disappearance, which may represent transient electrophysiological stunning (reversible electroporation) rather than irreversible myocardial ablation. As such, acute arrhythmia suppression or non-inducibility may potentially result from reversible electroporation effects within the targeted substrate, rather than permanent lesion formation. Thus, traditional procedural endpoints, such as EGM elimination, non-capture with high output pacing, noninducibility, and the use of waiting periods to confirm lesion durability, may be less reliable when applied to PFA. These limitations highlight a gap in our current understanding of lesion durability in ventricular PFA and underscore the need for alternative validation strategies.

While feasibility and procedural safety are becoming increasingly evident, the question of true effectiveness, defined as durable substrate modification and sustained arrhythmia control, remains to be fully established.

#### Lesion characterization from animal and *in vivo* data

Experimental studies consistently suggest that PFA lesion formation is less attenuated by scar than RF ablation and may also traverse tissue interfaces containing collagen or fat more effectively.^1^ In a swine model of healed myocardial infarction, PFA created more homogeneous and better demarcated lesions than RF, with greater depth and a higher rate of transmurality.^54^ Lesion extension through collagen and fat towards the epicardium with preservation of intramural blood vessels or nerve bundles was observed with PFA but not with RF. Other animal work confirmed that lesion depth in scarred myocardium may still be somewhat smaller than in healthy myocardium but remains substantial and generally superior to RF in infarct substrate.^54–56^ Additional work demonstrated penetration of both infarct and iatrogenic scar, with depths up to 8.6 mm in some sections, while QRS-gated delivery eliminated ventricular arrhythmias that were occasionally provoked by ungated pulse delivery.^56^

Another important observation from preclinical work is the repetition dependence for lesion depth. Repeated applications increase lesion depth and size, which may be particularly important in thick myocardium or complex structures (e.g. septum, papillary muscles).^57,58^ In ventricular models, this approach has achieved lesion depths of 6–9 mm and, in bipolar configurations, transmural lesions exceeding 14 mm without overt thermal injury.

#### Catheter configurations, feasibility, safety, and effectiveness

##### Small-footprint focal PFA systems

The earliest structured clinical data for ventricular PFA come from focal PFA delivered through contact force-sensing catheters. PFA was feasible across multiple ventricular locations to treat premature ventricular contractions (PVCs), with acute and chronic success both reported at 85%.^59^ Irritative ventricular firing occurred in 55% of patients; of note, acute remapping findings suggested that at least some early lesions included a reversible component rather than immediate permanent substrate elimination.

A broader two-centre ventricular arrhythmia experience including both PVC and scar-related VT confirmed that focal PFA is technically feasible and acutely effective, with an overall acute success of 84%.^60^ Yet this study also illustrated the difference between acute feasibility and true effectiveness. While 3-month suppression of PVCs remained good at 81%, only 52% of patients with scar-related VT were free of recurrent VA at follow-up. In addition, three patients developed transient conduction system block due to unexpected electric current leakage from proximal shaft electrodes, underscoring that purpose-built catheter design, not just waveform, critically affects safety.

The most recent major development is the introduction of high-voltage focal PFA systems designed specifically for scar-related VT.^61^ The first-in-human VCAS study represents a strong clinical signal that ventricular PFA is moving towards meaningful substrate ablation. In 26 patients with scar-related VT, acute procedural success was achieved in 92%, 6-month freedom from recurrent VT or ventricular fibrillation (VF) or Implantable Cardioverter Defibrillator (ICD) shock was 81.8%, and VT/VF burden fell by 98%. Most importantly, in a subgroup undergoing paired endocardial and epicardial mapping, transmural tissue homogenization after endocardial-only PFA was acutely observed in all evaluated patients.

##### Large-footprint focal PFA systems

The next step in the evolution of ventricular PFA has been the development of large-footprint catheters (see *Figure 2*), which may permit high-density mapping and delivery of RF and/or PFA energy. Initial small studies showed that this strategy is feasible in complex ventricular substrate and may enable faster and broader substrate modification than focal catheters.^62,63^ In an early-feasibility study of 18 patients with recurrent VT, 3-month ventricular-arrhythmia-free survival reached 78%; however, coronary spasm occurred in most patients undergoing epicardial PF delivery, although all episodes resolved with nitrates.^63^

**Figure 2:**
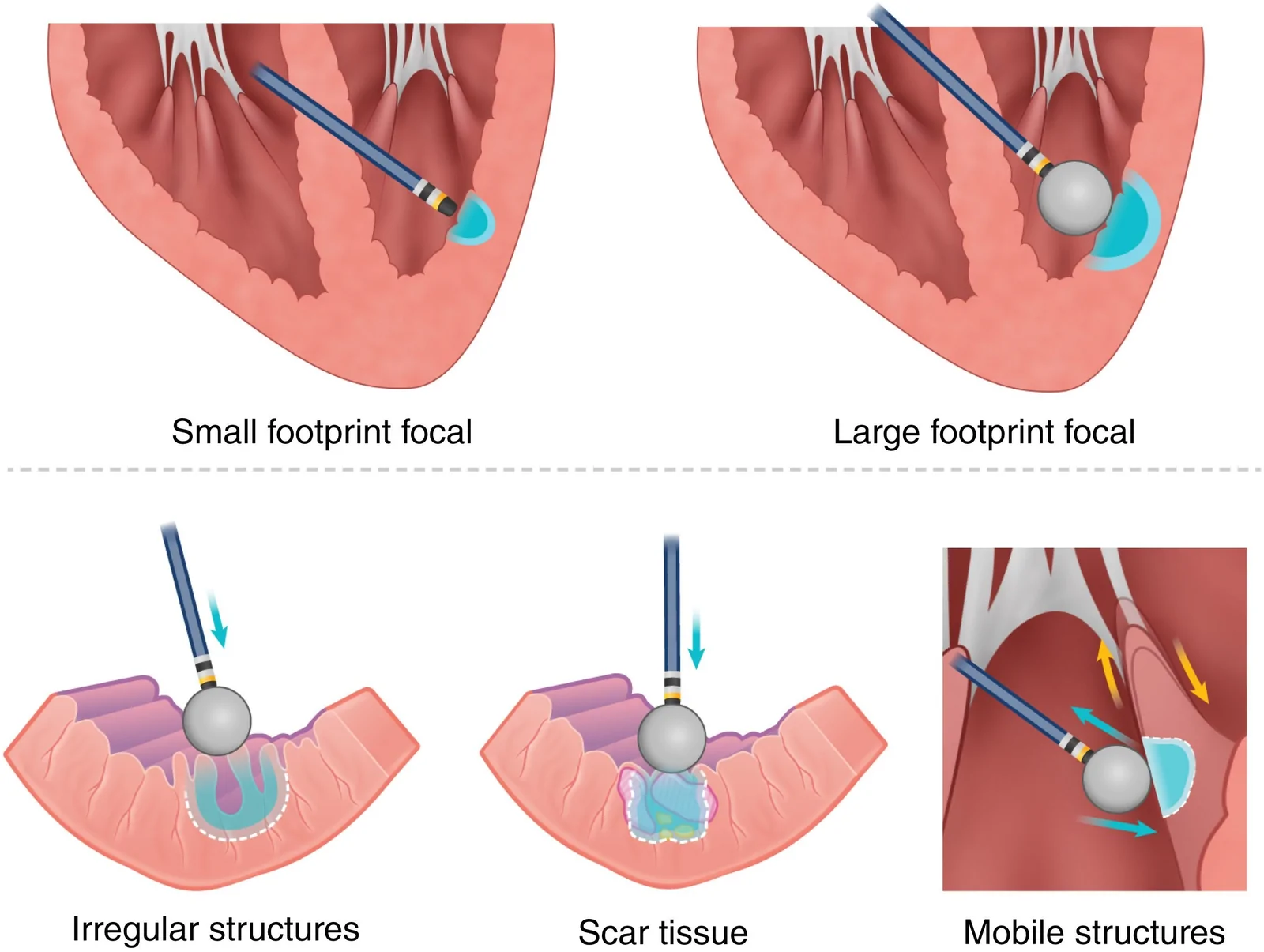
PFA applications in the ventricle.

The multicentre AVAAR registry provided the first larger-scale real-world assessment of a large-footprint focal PFA system. Among 126 patients, acute success was 84% and arrhythmia-free survival at a mean of 5.6 months was 72%.^64^ These outcomes support procedural feasibility and suggest reasonable short-term efficacy in a difficult population. However, this registry also reported safety events: major complications occurred in 6%, including thromboembolic events and one ventricular fibrillation episode related to catheter insulation breach, while minor complications included transient ST changes, haemolysis, mechanical left bundle branch block, and chordal injury without sequelae. Overall, large-footprint focal systems may potentially improve feasibility by enabling rapid mapping and wider lesion sets, but their true effectiveness still depends on whether the created lesions are truly transmural and durable, especially in non-ischaemic cardiomyopathy where trabeculation, variable wall thickness, and intramural substrate remain major obstacles.

#### Future perspectives

Reversible electroporation offers a compelling, still largely exploratory concept for arrhythmia mapping by enabling transient and localized electrical silencing of arrhythmogenic substrate without causing permanent tissue injury.^52,65^ This strategy has the potential to evolve into a novel functional mapping tool for VT (*Figure 3*), bridging the gap between substrate identification and ablation; however, its role will depend on improved control of energy delivery and validation in dedicated clinical studies.

**Figure 3:**
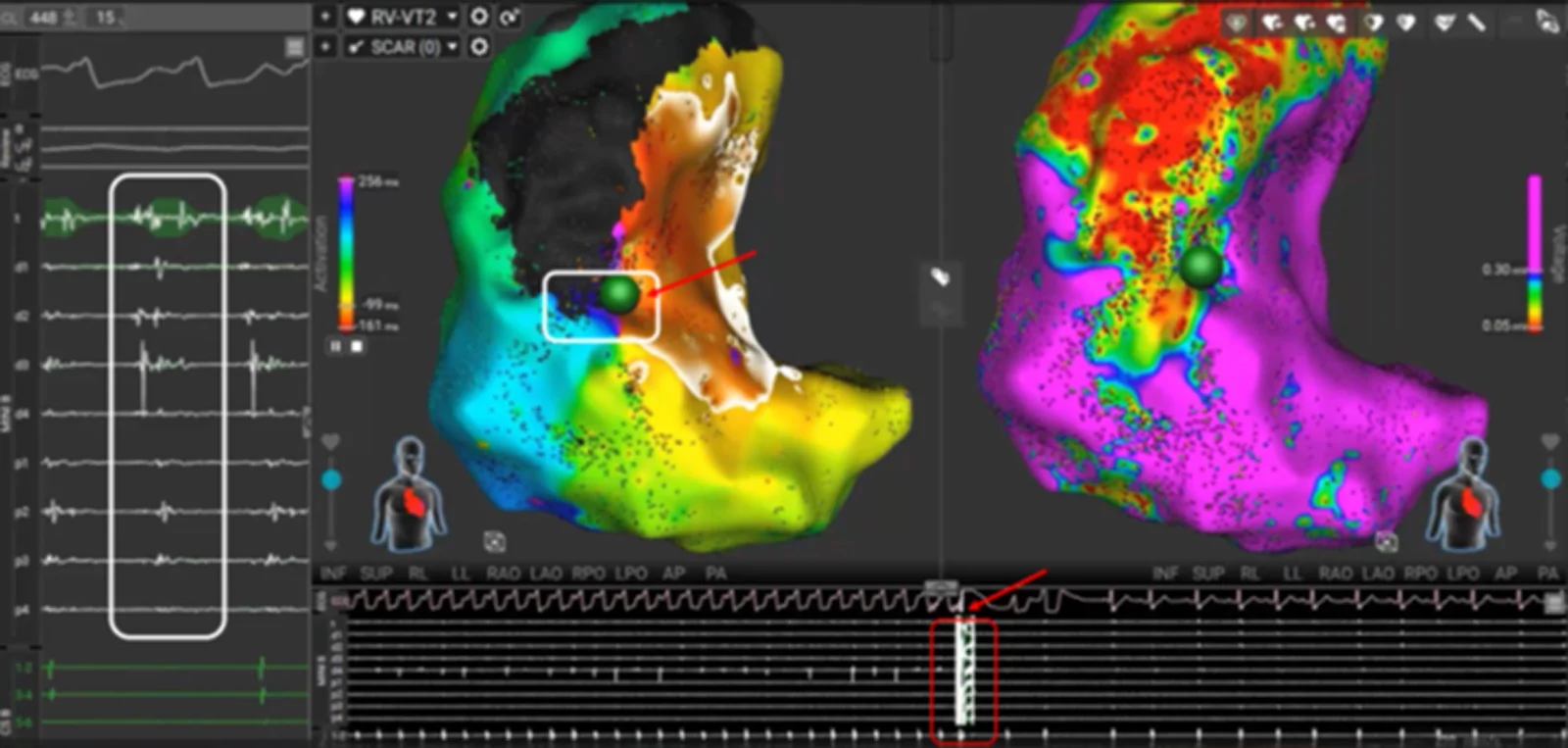
Reversible electroporation for ventricular arrhythmia mapping. The VT activation map (left panel) demonstrates a figure-of-eight re-entry pattern, with mid-diastolic potentials identified at the VT isthmus (white rectangle). Application of reversible electroporation at this critical isthmus (red arrow) results in termination of the VT.

Future progress in ventricular PFA will also depend on establishing reliable procedural endpoints that accurately reflect durable lesion formation rather than transient electrophysiological effects. In parallel, optimization of energy delivery and dosing strategies, together with the development of dedicated catheter designs specifically engineered for ventricular anatomy and substrate complexity, will be essential to enable tailored PFA titration for different tissue characteristics and to consistently achieve sufficient lesion depth and transmurality.

### New patient groups

Pulsed field ablation signifies one of the most substantial innovations in the field of cardiac electrophysiology, with an unprecedented adoption into clinical practice largely because of advantages in procedural efficiency and safety when compared to thermal ablation. In addition to PFA becoming the preferred initial ablation modality for paroxysmal and persistent AF, there has been an organic reappraisal of patient groups being offered intervention. This reappraisal has centred on the concept of the risk-benefit ratio of intervention, whereby clinicians and patients balance the anticipated magnitude of benefit against the anticipated magnitude of risk associated with intervention, an analysis that is unique to various patient groups (see *Figure 4*).

- **Asymptomatic patients.** It is estimated that more than one third of patients with AF are asymptomatic. Patients with asymptomatic AF have been considered to be a group with a lower perceived benefit of intervention, as the core benefit of catheter ablation is improvement in symptoms and quality of life, and a group who is at lower risk of adverse AF-associated complications. However, large studies have demonstrated comparable rates of all-cause mortality, cardiovascular mortality, and stroke/systemic embolism in those with symptomatic and asymptomatic AF,^66^ and recent interventional studies have demonstrated that the benefit of early rhythm control was maintained in the absence of symptoms (hazard ratio for primary outcome in asymptomatic patients of 0.76, 95% CI 0.57–1.03 vs. 0.79 in symptomatic patients, 95% CI 0.64–0.98).^67^ This suggests that an intervention with a low magnitude of risk (e.g. PFA) may be beneficially employed in asymptomatic patients to improve clinical outcomes, with consideration of patient preferences as part of the shared decision-making process.
- **Elderly patients.** Elderly patients (≥ 75 years) represent a high-risk subgroup, in terms of adverse AF-associated cardiovascular outcomes (e.g. death, disabling stroke, major bleeding, and cardiac arrest)^68,69^ and adverse procedural outcomes (higher rates of AF recurrence and higher risk of interventional complication).^70^ However, PFA appears to be associated with a beneficial risk-benefit ratio, as PFA is not associated with reduced efficacy in the elderly subgroup (e.g. preserved benefit) and confers a lower risk of procedure-related complication (e.g. reduced risk). As such, in contrast to thermal ablation, it can be expected that PFA will be offered to an increasing number of elderly patients.
- **Patients at elevated procedural risk.** Patients with increasing numbers of cardiovascular comorbidities are often not offered invasive intervention out of concern of prohibitive procedural risk. In contrast to thermal ablation, PFA is capable of minimally thermal and selective tissue injury, minimizing the risk of collateral damage (e.g. to the phrenic nerve or oesophagus) and avoiding the negative effects of thermal energy (e.g. coagulum formation or pulmonary vein stenosis). As such, PFA offers a novel opportunity for invasive intervention for the subset of patients considered too high risk for complications related to thermal ablation.
- **Structural atrial pathology and longstanding persistent AF**. Patients with more advanced atrial structural pathology are known to manifest more advanced forms of AF (e.g. longstanding persistent AF) and experience an inferior freedom from recurrent atrial tachyarrhythmia compared to ablation for paroxysmal atrial fibrillation (e.g. attenuated benefit of intervention).^41^ Moreover, patients with atrial myopathy and more persistent forms of atrial fibrillation have a greater burden of comorbidities and experience higher rates of peri-procedural complications (e.g. increased risk of intervention).^71^ In this subgroup PFA offers a potential efficacy advantage^72,73^ as it is able to better penetrate and homogenize the scarred myocardium associated with atrial myopathy.^74^ Given the favourable efficacy and safety profile associated with PFA, it can be expected that PFA will be increasingly offered to patients with structural atrial pathology and more advanced forms of AF.
- **Concomitant AF and left atrial appendage occlusion**. The OPTION trial demonstrated the benefit of combined AF ablation and left atrial appendage occlusion (LAAO) for patients at high risk of stroke.^75^ While these procedures were most often staged, there is increasing interest in the more patient-centric concomitant AF ablation and LAAO. When concomitant procedures are performed with thermal energy, there is a risk of significant oedema in the left atrial appendage-pulmonary venous ridge, which can lead to early LAAO device migration or a later peri-device leak. PFA causes less tissue swelling than thermal ablation, suggesting it may be the preferred energy modality for concomitant LAAO.

**Figure 4:**
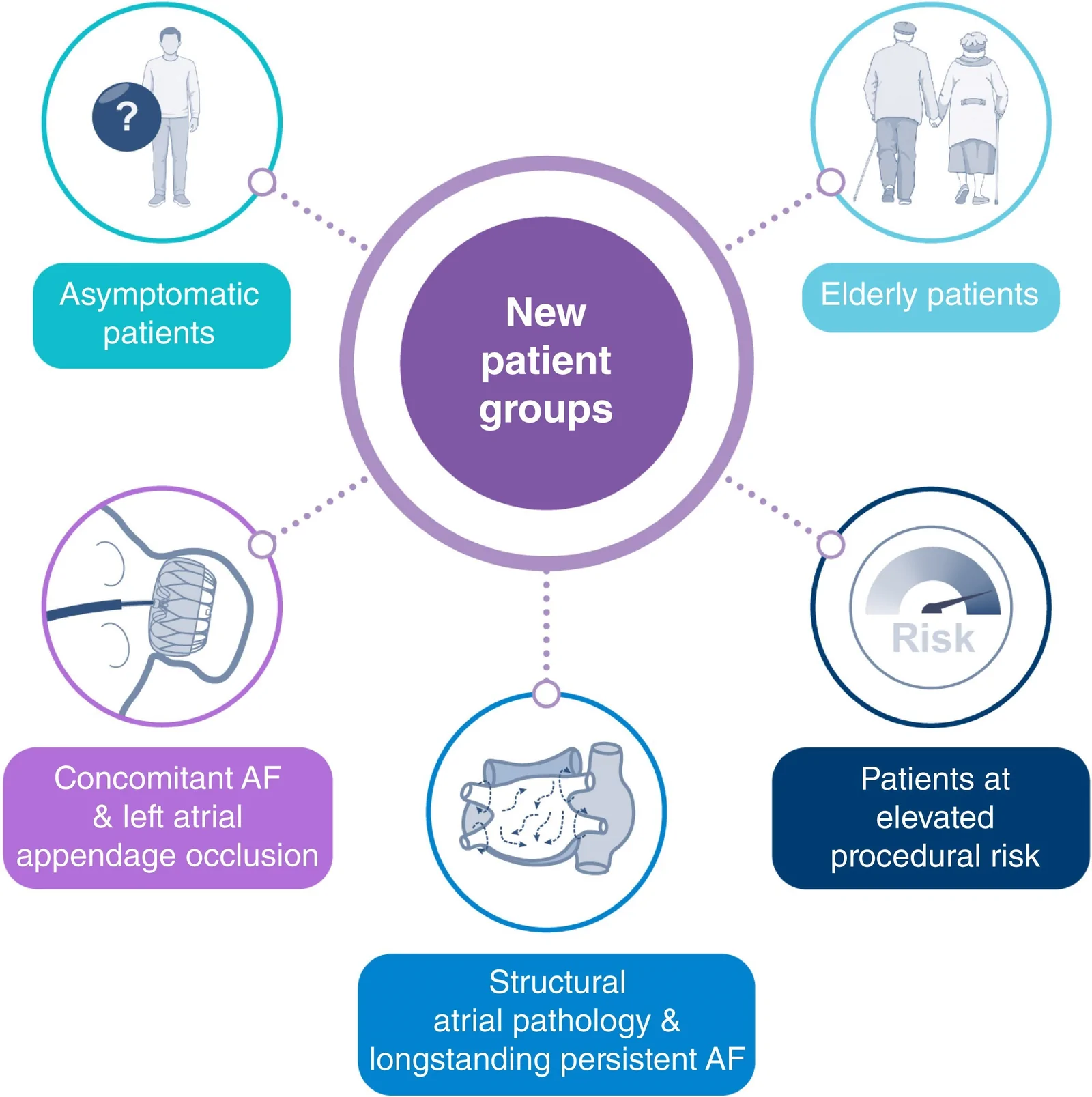
Summary of main new patient groups for future PFA applications.

## Remaining challenges in PFA: scientific and clinical aspects

### Knowledge gaps: unresolved questions in PFA

PFA has progressed rapidly from biophysical concept to clinical routine, driven by procedural efficiency and a favourable collateral profile. However, several scientific and clinical uncertainties remain, and these gaps may become more consequential as PFA expands beyond PVI.^51^

A core unresolved issue is the definition of ‘dose’ in PFA and how delivered parameters translate into durable tissue injury. Lesion formation reflects interactions between waveform, electrode geometry, tissue thickness, and catheter–tissue coupling; a uniform dosing paradigm to compare different PFA systems is unlikely. Current pulse protocols have been primarily developed and validated for antral PVI, whereas systematic dosing for extended atrial targets and other chambers is still evolving.^18^ Repeated applications are often used to increase lesion depth and width, but the dose–response relationship and the possibility of dose titration for durable transmurality remain incompletely defined.^18^ Accurate real-time *in vivo* field distribution remains difficult to predict, as local anatomy and tissue anisotropy may alter electric-field penetration and lesion geometry, particularly in thicker regions and for linear block.^18,51,76^

A second gap concerns intra-procedural assessment of lesion creation. It is not yet possible to reliably distinguish long-term failure due to incomplete target ablation from failure driven by reversible electroporation or acute reversible effects.^51^ Transient conduction impairment and reversible effects further complicate endpoint interpretation, and validated real-time markers that predict chronic durability are still lacking when broader atrial coverage and linear block are pursued. Lesion heterogeneity at borders may leave functional gaps that are not apparent on acute testing.^18,51^ Furthermore, the dynamics of recovery of reversible effects are not yet accurately defined and the validity of a universal optimal waiting period for all PFA systems is yet to be established.^32,35^

Long-term tissue response warrants further characterization. While preferential myocardial effects are supported, precise cardiomyocyte death mechanisms remain incompletely defined. The balance between acute cell death, triggered by catastrophic membrane disruption in areas receiving the highest PFA dose, and delayed cell death processes, including apoptosis and pyroptosis induced by calcium overload, ATP depletion, mitochondrial injury, and related mechanisms in lower-dose regions, remains poorly defined. A better understanding of these differential cell death pathways may provide important insights into lesion maturation, healing kinetics, and the evolution of post-ablation inflammatory responses. Additionally, the impact of repeated PFA exposures during consolidation or redo procedures remains incompletely defined across different atrial substrates and scar composition. Whether PFA produces distinct chronic scar architecture—and how this relates to organized atrial tachycardias—remains insufficiently characterized.^18,51^

Energy-related bioeffects represent an additional gap that becomes more relevant with broader use. Haemolysis and microbubble formation can arise from electrode–blood interactions and waveform-dependent electrochemical or thermal processes; importantly, no quantitative clinical threshold linking haemolysis severity to acute kidney injury has been established.^77^ Clinical studies indicate that PFA-induced haemolysis is common but usually subclinical. Plasma-free haemoglobin levels typically peak in the range of 30 to 60 mg/dL after AF ablation procedures, although higher values may occur with increasing numbers of pulse application.^78,79^ Microbubble generation is shaped by waveform properties, supporting the concept that waveform optimization may be required as indications broaden.^77,80^ Effects on coronary arteries, especially delayed events although very rare need more mechanistic studies to establish the real impact in clinical practice.^81^ Additionally, the role of combining different energy sources for increasing therapeutic effects also remains to be proven as current evidence is limited to a single preclinical study.^82^

Knowledge gaps also persist in special patient groups (i.e. populations under-represented in pivotal datasets). For example, in patients with cardiac implantable electronic devices, electrical field interactions with leads and generators remain insufficiently studied, and more systematic peri-procedural and long-term device evaluation is required.^83^

Finally, device and waveform innovation is outpacing biological validation. Many commercial waveforms remain non-disclosed, limiting mechanistic comparison and systematic optimization.^51^ Computational work suggests that catheter geometry can alter current density and lesion precision, but optimization remains challenging without chronic lesion correlation and greater transparency of pulse protocols.^84^ Reversible electroporation concepts illustrate the opportunity—and uncertainty—of using sub-lethal pulsing for functional mapping before irreversible lesion delivery; early experience suggests feasibility, but broader evaluation is required.^18^

Overall, addressing the existing knowledge gaps will require sustained efforts spanning both fundamental research and clinical investigation. While understanding of PFA has advanced considerably in recent years, unresolved issues continue to position it as a dynamic and promising area for future research and innovation. Ongoing collaboration among academic researchers, clinical specialists, and industry partners will be essential to ensure steady and well-integrated progress in the field.

### The need for a common language: standardization of PFA systems and reporting

The rapid adoption of PFA has ushered in a ‘bioelectric’ revolution in cardiac electrophysiology demonstrating high procedural efficiency and a favourable safety profile compared to thermal modalities. However, the rapid implementation has outpaced the development of standardized definitions and reporting frameworks resulting in significant heterogeneity in how PFA systems, parameters and procedural endpoints are described and reported across studies and clinical reports.^85^ This ambiguity makes it difficult to synthesize data and establish long-term clinical benchmarks for this maturing technology.

#### Why is standardization challenging in PFA?

Standardization is primarily hindered by the proprietary and often undisclosed nature of PFA waveforms.^84,86^ Manufacturers utilize distinct ‘pulse recipes’ or profiles that vary widely in pulse amplitude, width (ranging from nanoseconds to milliseconds), numbers, frequency, and waveform polarity (monophasic vs. biphasic).^18^ Furthermore, diverse catheter architectures, ranging from over-the-wire circular arrays to focal lattice-tip and ‘balloon-in-basket’ designs, drastically alter the resulting electric field distribution and energy density in tissue.^18^ Some designs may even exhibit an electric field shielding effect in certain tissue areas potentially requiring more electric current delivered.^84^ Unlike RF ablation, where ‘Power’ and ‘Duration’ are universal metrics, PFA lacks a unified dose metric that accounts for these complex waveform and hardware interactions.^87,88^

#### Why is it needed?

Standardization is essential for comparability across platforms and clinical trials.^86^ Without harmonized reporting, clinicians cannot determine if varying outcomes or complication rates are inherent to PFA as a modality or specific to a particular waveform or catheter footprint. For example, side effects such as haemolysis (which appears dose-dependent) or coronary spasm are recognized as PFA class effects, but lack of standardization in reporting of clinically significant thresholds makes system comparison difficult. Transparency is also critical because PFA systems are constantly evolving. Moreover, standardization is critical for the development of multimodal clinical registries that leverage AI to interpret data across different institutions.

#### Common mistakes and misleading concepts

Several misconceptions in PFA extend beyond theoretical misunderstandings and influence how results are reported, interpreted and compared across studies.

The most prevalent reporting mistake in studies is relying on acute isolation and signal loss as a surrogate for durable lesion formation.^35^ While the disappearance of bipolar electrograms is a standard endpoint in thermal ablation, in PFA it often reflects ‘cellular stunning’ or reversible electroporation rather than permanent cell death.^18,35^ Emergent research suggests that unipolar signals may be a more reliable indicator of durable irreversible lesions than bipolar signal elimination. Recent experimental and clinical studies suggest that the most informative unipolar electrogram markers following PFA are abolition of the terminal S-wave, reduction of unipolar voltage to ≤0.4 mV, prominent ST-segment elevation, and dynamics of these abnormalities over time.^32–35^ Finding reliable acute endpoints to report in PFA remains an open challenge. As a result, acute procedural success is systematically overestimated.

A second major misconception is the implicit assumption that ‘PFA is PFA’, leading to aggregation of results across fundamentally different systems. In reality, lesion formation depends on electric field distribution, pulse amplitude, duration, number of pulses and trains, polarity and vectoring, catheter geometry and electrode configuration. These parameters are highly system-specific and often proprietary, resulting in markedly different biological effects, including lesion depth and safety profiles.

Another misleading concept is that PFA is completely non-thermal^74^ or independent on catheter-tissue contact, contributing to inconsistent reporting of safety endpoints and procedural variables. From a reporting standpoint, this misconception leads to a lack of standardized monitoring of surrogate markers such as microbubbles, temperature rise and difficulty attributing complications such as thromboembolism or coagulation to specific mechanisms.

Collectively, these misconceptions do not simply reflect gaps in mechanistic understanding but lead to heterogeneous and often non-comparable datasets.

#### Finding a common language

To find a common language, recent consensus guidelines propose a harmonized nomenclature^85,86^:

- PFA profile: The specific, disclosable pulse parameters of a system (such as amplitude, width, and shape).
- PFA application: A single successful delivery of all programmed pulse trains at a single catheter position.
- Catheter classification: Defining catheters as focal (≤4 mm tip), large-tip focal (4–12 mm), or regional (4–12 mm), or regional (>12 mm) to better characterize their tissue footprint.

Committees now call for minimal technical disclosure in all published manuscripts, including pulse amplitude, vectoring (monopolar vs. bipolar), duty cycle and total energy delivered. Only through such transparency and the use of protocol-mandated remapping studies for new devices can the field transition from empirical workflows to a predictable and standardized PFA therapy.

### System comparison: why is comparing PFA systems so hard?

Why is comparing PFA systems so hard—but still so important for future scientific progress as well as better safety and procedure outcomes?

As described previously, there are numerous variations among the different PFA systems regarding PFA waveform, energy delivery pattern, dosing, catheter characteristics, available intraprocedural feedback information, surrogates for lesion durability, etc.^51,86,89^ Furthermore, design variables such as waveforms are continuously optimized while this information is not publicly available among PFA systems. Even if manufacturers disclosed waveform characteristics, each waveform is intrinsically linked to a specific catheter design, preventing direct comparison between different waveforms within a standardized experimental model.

Reliable biological models for acute assessment of lesion formation, particularly for distinguishing reversible from irreversible electroporation and evaluating PFA durability, are limited, as PFA effects require an intact *in vivo* system and sufficient time for lesion maturation. Acute changes in local electrograms and conduction properties, such as signal attenuation or conduction block, are not reliable procedural endpoints limiting the feasibility of comparing different PFA systems in simple experimental models.

The above-mentioned factors make evaluation of acute treatment effects as well as direct comparison between PFA systems very difficult at this stage, representing a key limitation of the technology. A potential way forward is to develop a standardized framework that enables comparison of PFA systems based on available device data and shared evaluation criteria.

Availability of PFA waveform and energy information as well as common standards for energy description and delivery should be a mandatory first step forward.^86^ Although this information alone would not be enough, it could be a first step for developing a basic model for acute system comparison. Next step will be further development of *in vitro* models and surrogates for measuring *in vivo* safety, tissue-effects and lesion durability.

From a clinical perspective, while standarized metrics are currently under investigation, some procedural parameters could be proposed for system comparison. Potential acute surrogate endpoints for durable electroporation and clinical response include distribution of applied electric field, tissue proximity indicators, local unipolar and bipolar post-ablation signal changes, impedance response or a combination of these factors. However, the underlying details of these parameters are typically not disclosed for each system, which again complicates direct comparison. In addition, patient-specific factors, such as differences in cardiac anatomy, target tissue thickness and local fibrosis, cause a variable individual response to PFA and to different PFA systems in both healthy and abnormal hearts.

Lastly, very limited data are available directly comparing different PFA systems in head-to-head studies looking at long-term clinical endpoints, such as freedom from arrhythmias. Therefore, comparison of different PFA systems is now based on data from different studies that are not directly comparable. Long-term head-to-head comparisons including procedural safety, remapping data to assess lesion durability and comparison of clinical outcomes should be prioritized. Safety parameters may include risk of clinically significant haemolysis, vasospasm, significant thrombus and stroke formation, as well as the risk of malignant arrhythmias. Outcome parameters may include both biomarkers, noninvasive imaging, patient-reported outcomes and arrhythmia recurrence.

The EP community together with the industry are encouraged to combine forces to enable meaningful comparison of different PFA technologies. Only then is it possible to further optimize PFA energy delivery and get safe and durable lesion formation. The ultimate aim is to improve clinical outcomes consistently, regardless of patient characteristics, operator, or ablation system.

### Clinical trials: what future studies must address?

So far, clinical evidence in PFA can be considered relatively mature for initial PFA systems while robust data is still under development for more recently approved systems. Large registries and pivotal trials on AF ablation with PFA show high procedural success rates (i.e. PVI) with low major complications (0.7–2%) and 1-year freedom from atrial arrhythmia for paroxysmal AF between 65 and 85% approximately, depending on the system studied.^47,90–94^ It is important to highlight that cross-trial comparison is limited by differences in efficacy definitions, monitoring intensity, inclusion of antiarrhythmic drug escalation or repeat ablation in composite endpoints.

Recent trials have been designed aiming at indication extension to cover the broad spectrum of AF: since the first pivotal trial of a PFA technology for the treatment of drug-refractory paroxysmal AF in 2024,^95^ studies have been published covering also drug-refractory and first-line persistent AF (ADVANTAGE-AF, phases I and II, NCT05443594, PULSED AF NCT04198701, VOLT-AF NCT06223789, SPHERE Per-AF NCT05120193, AVANT-GUARD study NCT06096337).^72,73,91,96–98^ Ongoing research is extremely active with a focus on indication expansion, managing repeat procedures, assessing new catheter technologies and performing head-to-head comparisons with traditional thermal ablation, yet there are still several domains where PFA clinical evidence requires further research. The extension to more complex forms of AF brings the need to assess the safety and performance of additional lesion sets beyond PVI: while ablation of the posterior wall with PFA has been already studied in recent trials,^73^ assessment of other lesions, which are common to the treatment of persistent AF or repeat ablations, such as the mitral isthmus, LA roof or superior vena cava still needs to be comprehensively assessed through upcoming clinical trials. Additionally, the development of focal and large-focal PFA catheters allows the creation of linear lesions to manage supraventricular tachycardias (SVT) such as cavo-tricuspid isthmus (CTI)-dependent atrial flutter and macro-reentrant atrial tachycardias. For SVTs other than CTI-dependent flutter, only small series have been published to date, showing very high acute success (98%) and 6-month recurrence of 10%,^99^ while a more complete assessment of these arrhythmias should be integrated in future pivotal trials.

From the safety perspective, PFA has demonstrated a favourable profile so far, generally outperforming conventional thermal ablation. However, some particular effects such as haemolysis and coronary spasm, although rarely associated with clinically relevant adverse events, need a full understanding in this context. Current studies are thoroughly assessing the real occurrence of such complications and studying procedure-specific mitigation strategies. For the prevention of renal failure, protocols are investigating dedicated hydration workflows to be undertaken in the periprocedural setting. For coronary vasospasm, upcoming clinical studies should better assess through imaging acute and potential chronic extent of this phenomenon with PFA but also with traditional thermal energy.

From the effectiveness perspective, attention should be given both to long-term follow-ups and, where possible, to remapping data to assess lesion durability. Monitoring strategies are also evolving with the use of implantable cardiac monitors (ICM) that allow continuous retrieval of arrhythmia recurrence information without the need for scheduled in-clinic follow-ups, facilitating long-term follow-ups especially after one year. Use of ICM has become a standard for new IDE studies, opening the pathway to use AF burden-based outcomes, and link burden thresholds to clinical events that may constitute a paradigm shift in the overall management of AF. The first long-term follow-up publication for PFA (up to 4 years) was recently reported for the pentaspline catheter.^100^ On the other hand, lesion durability assessment through 3D remapping procedure should be part of every first-in-human clinical study and will likely remain a key point of trial design as PFA technology is optimized. Lesion durability data will also be informative for dosing optimization, which is considered important for the consolidation of standardized protocols to maximize effectiveness.^18,44,101–103^

The role of improved imaging techniques such as intracardiac echocardiography and advanced 3D mapping to support PFA procedures is also an important field where upcoming studies should focus. Integrating novel 3D mapping tools with PFA may enhance procedural precision, but future studies should establish the impact on long-term outcomes. In particular, the integration of advanced mapping tools such as electrographic flow mapping is object of ongoing randomized clinical studies evaluating this novel, personalized approach for treating persistent AF in combination with PFA.^104^ Further exploration of similar approaches with focal catheters in complex cases such as repeat ablation will allow assessing if personalized therapy planning can improve long-term outcomes and efficiency.

Lastly, PFA therapy of ventricular arrhythmias is a promising field of application. To date, several prospective cohorts of patients have been studied showing good acute success.^60,61,64,105,106^ Lesion depth and durability, long-term effectiveness and complication assessment will be key elements to evaluate in upcoming clinical studies to better assess the value of this technology in this area.

## Conclusions

While PFA has rapidly transformed the cardiac electrophysiology field, the future ahead of this technology remains promising and will be shaped by ongoing technological refinement, expansion into broader and more complex arrhythmia indications and advancements in research to close current mechanistic knowledge gaps. Although PFA is increasingly regarded as the leading ablation technology of the future, it remains uncertain whether it will completely replace conventional thermal energy sources.

Despite its rapid clinical adoption, significant challenges remain in PFA, particularly regarding the standardization of efficacy and safety endpoints and cross-system comparison across different technologies. Another important challenge in the evolving PFA landscape is education within the electrophysiology community. The novel concepts associated with PFA mechanisms and platform-specific procedural considerations will need to be progressively incorporated into future electrophysiology training programmes and standardized educational pathways.

PFA has reshaped the landscape of catheter ablation, but its long-term consolidation will depend on continued technological refinement, rigorous long-term evaluation, and careful integration into evidence-based clinical practice.

## Data Availability

The data underlying this article will be shared on reasonable request to the corresponding author.
